# Role of Central Serotonin in Anticipation of Rewarding and Punishing Outcomes: Effects of Selective Amygdala or Orbitofrontal 5-HT Depletion

**DOI:** 10.1093/cercor/bhu102

**Published:** 2014-05-30

**Authors:** Rafal Rygula, Hannah F. Clarke, Rudolf N. Cardinal, Gemma J. Cockcroft, Jing Xia, Jeff W. Dalley, Trevor W. Robbins, Angela C. Roberts

**Affiliations:** 1 Department of Psychology, University of Cambridge, Cambridge CB2 3EB, UK; 2 Behavioural and Clinical Neuroscience Institute, University of Cambridge, Cambridge CB2 3EB, UK; 3 Department of Physiology, Development and Neuroscience, University of Cambridge, CambridgeCB2 3DY, UK; 4 Department of Psychiatry, University of Cambridge, School of Clinical Medicine, CambridgeCB2 0QQ, UK; 5 Liaison Psychiatry Service, Cambridgeshire and Peterborough NHS Foundation Trust, Box 190, Addenbrooke's Hospital, Cambridge CB2 0QQ, UK; 6 Current Address: Affective Cognitive Neuroscience Laboratory, Department of Behavioral Neurobiology and Drug Development, Institute of Pharmacology Polish Academy of Sciences, ul Smetna 12, 31-343 Krakow, Poland

**Keywords:** amygdala, marmoset, orbitofrontal cortex, probabilistic reversal learning

## Abstract

Understanding the role of serotonin (or 5-hydroxytryptamine, 5-HT) in aversive processing has been hampered by the contradictory findings, across studies, of increased sensitivity to punishment in terms of subsequent response choice but decreased sensitivity to punishment-induced response suppression following gross depletion of central 5-HT. To address this apparent discrepancy, the present study determined whether both effects could be found in the same animals by performing localized 5-HT depletions in the amygdala or orbitofrontal cortex (OFC) of a New World monkey, the common marmoset. 5-HT depletion in the amygdala impaired response choice on a probabilistic visual discrimination task by increasing the effectiveness of misleading, or false, punishment and reward, and decreased response suppression in a variable interval test of punishment sensitivity that employed the same reward and punisher. 5-HT depletion in the OFC also disrupted probabilistic discrimination learning and decreased response suppression. Computational modeling of behavior on the discrimination task showed that the lesions reduced reinforcement sensitivity. A novel, unitary account of the findings in terms of the causal role of 5-HT in the anticipation of both negative and positive motivational outcomes is proposed and discussed in relation to current theories of 5-HT function and our understanding of mood and anxiety disorders.

## Introduction

Serotonin (5-hydroxytryptamine, 5-HT) has long been implicated in aversive processing but its precise role remains unclear (Cools, Roberts et al. 2008). This is due, in part, to the opposing effects that global depletions of 5-HT appear to have on punishment processing. Increases in sensitivity to punishment have been reported in contexts involving behavioral choice (Evers et al. 2005; Cools, Roberts et al. 2008; Cools, Robinson et al. 2008; Roiser et al. 2008), while diminishing effects of punishment have been reported on the suppression of responding (Robichaud and Sledge 1969; Graeff and Schoenfeld 1970; Tye et al. 1977; Soderpalm and Engel 1991; Clarke et al. 2004; Boulougouris et al. 2007). Resolving these conflicting results is important not only in view of the ambiguous status of altered 5-HT neurotransmission in depression and anxiety and its amelioration with serotonergic agents (Deakin and Graeff 1991; Massart et al. 2012), but also in guiding theoretical accounts of 5-HT function. Moreover, it is also important to determine whether the effects of 5-HT are specific to punishment processing or extend into the reward domain. Patients with depression have been shown to be particularly sensitive to spurious negative feedback (Elliott et al. 1996; Murphy et al. 2003) and restoring the euthymic balance by correcting 5-HT transmission may constitute a primary mode of action of antidepressant drugs. However, whether this restoration arises from reductions in sensitivity to punishment and/or direct positive effects on reward function is unclear.

Previously, we hypothesized that such contrasting effects of punishment on behavioral suppression and its subsequent impact on choice behavior could be due to the differential role of 5-HT transmission at cortical and subcortical levels (Cools, Roberts et al. 2008). In particular, we hypothesized that 5-HT activity might directly influence aversive processing subcortically, for example, in the amygdala, whereas 5-HT modulation of orbitofrontal cortical function might bias descending inhibitory control of subcortical mechanisms, including the expression of emotional processing in the amygdala and behavioral output in the striatum. Alternatively, the contrasting effects could be due to the dissimilar procedures used to reduce 5-HT in humans and animals (which may differentially affect phasic and tonic modes of neurotransmission), or the differing behavioral tasks investigated, placing demands on distinct aspects of performance, such as inhibitory control (Clarke et al. 2004; Boulougouris et al. 2007) or aversive processing (Cools, Roberts et al. 2008).

To resolve these issues, we directly compared the effects of marked localized 5-HT depletion within the orbitofrontal cortex (OFC) and amygdala to determine whether these contrasting effects could be dissociated neuroanatomically or would occur together, suggesting a common origin. A New World primate, the common marmoset, received 5,7-dihydroxytryptamine (5,7-DHT)-induced depletions of 5-HT prior to evaluation on two tests that used the same punishing stimulus to measure sensitivity to negative feedback and punishment-induced suppression of responding. For the former, we used a probabilistic visual discrimination learning and reversal (PVDLR) task in which subjects learnt, which of two discriminative stimuli was associated with the better outcome from rewarding feedback (5 s banana milkshake) and punishment (0.3 s mildly aversive 108 dB loud noise). For successful completion of the task, subjects had to learn to ignore infrequent and misleading negative (and positive) feedback, arising from the probabilistic (80:20 or 70:30) nature of the discrimination. This task is very similar to that already shown to be sensitive to 5-HT manipulations in humans (Murphy et al. 2002) and rodents (Bari et al. 2010). To measure punishment-induced suppression of responding, monkeys' response rates were compared on a variable interval (VI) schedule of reward in the presence or absence of response-contingent, mildly aversive loud noise (108 dB).

## Materials and Methods

### Subjects and Housing

Twelve experimentally naïve common marmosets (*Callithrix jacchus*; 5 females, 7 males) bred on site at the University of Cambridge Marmoset Breeding Colony were housed in pairs. All monkeys were fed 20 g of MP.E1 primate diet (Special Diet Services, SDS) and 2 pieces of carrot 5 days per week after the daily behavioral testing session, with simultaneous access to water for 2 h. At weekends, they received fruit, rusk, malt loaf, eggs, and treats, and had access to water ad libitum. Their cages contained a range of environmental enrichment aids that were regularly varied, and all procedures were performed in accordance with the UK Animals (Scientific Procedures) Act 1986 under project licence 80/2225.

### Apparatus

Behavioral testing took place in a specially designed, sound-attenuated box in a dark room. The animal was positioned in a clear, plastic transport box, one side of which was removed to reveal a color computer monitor (Samsung). The marmoset reached through an array of vertical metal bars to touch stimuli presented on the monitor, and these responses were detected by a series of infrared beams (Intasolve, Interact 415) attached to the screen. Banana milkshake (Nestlé), which served as a reward, was delivered to a centrally placed licking spout (licker) for 5 s. Mildly aversive loud noise (108 dB) that served as punishment was played through a loudspeaker located at the back of the testing box. The test chamber was lit with a 3-W bulb. The stimuli were presented using the Whisker control system (Cardinal and Aitken 2010) running Monkey CANTAB (designed by Roberts and Robbins; version 3.6) (Cardinal 2007) which also controlled the apparatus and recorded responses.

### Behavioral Training and Experimental Design

All monkeys were trained initially to enter a clear plastic transport box for marshmallow reward and familiarized with the testing apparatus. Monkeys then received the following sequence of training: familiarization with milkshake reward, learning a tone–reward contingency, and responding on the touch screen until they were reliably and accurately making 40 responses or more to a square stimulus presented to the left and right of the licker in 20 min. (For full experimental details, see (Roberts et al. 1988).) After preliminary behavioral training, the marmosets were tested in the experimental procedures.

#### Probabilistic Visual Discrimination with Reward and Punishment

This consisted of two-choice discriminations composed of abstract, colored stimulus patterns (32 mm wide × 50 mm high; centres 12 cm apart). For all discriminations, a pair of stimuli was presented to the left and right of the center of the screen. A response to the predefined correct stimulus resulted in the incorrect stimulus disappearing from the screen, and the availability of 5 s of reinforcement delivered to the licking spout. After a response to the incorrect stimulus, both stimuli disappeared from the screen, the house light was extinguished for a 5-s timeout period and a mildly aversive, loud noise (108 dB) was presented for 0.3 s. The reward and punishment contingencies in this task were probabilistic, ranging from 0.7 to 0.9. For example, on a 0.8 probabilistic discrimination, responses to the “correct” stimulus were rewarded on 80% of trials and punished on 20% of trials, while the inverse was the case for the “incorrect” stimulus. The intertrial interval was 3 s and, within a session, the stimuli were presented equally to the left and right sides of the screen. Each monkey was presented with 40 trials per day, 5 days a week, and progressed to the next discrimination after attaining a criterion of 90% correct in the immediately preceding session.

##### Before surgery

**Figure 1.**
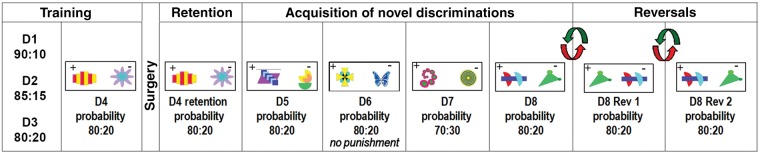
Probabilistic discrimination task. Illustrations of the stimuli used for each discrimination problem. D4 was performed immediately prior to surgery while all the other discriminations, including D4 retention, were performed after surgery.

Marmosets were presented with 4 probabilistic discriminations using a different pair of visual stimuli for each. During the first 3 discriminations, the probability was gradually changed, from 0.9 (discrimination 1), through 0.85 (discrimination 2) to 0.8 (discrimination 3). During these early stages, the mildly aversive loud noise was presented at a reduced intensity of 80 dB. After reaching criterion on the third discrimination, animals were maintained on this discrimination, while the noise intensity was gradually increased from 80 to 108 dB. This resulted in a decline in performance in some animals and so all animals were maintained on this discrimination until they had regained criterion. After the fourth and final presurgery discrimination (new pair of stimuli, probability level 0.8, and aversive 108 dB noise; Fig. 1, “D4”), monkeys received 5,7-DHT lesions of the amygdala, OFC, or a sham control procedure.

##### After surgery

After the recovery period, the animals were presented with:

1. the final presurgery discrimination as a retention test (Fig. 1, “D4 retention”).
2. a series of 4 novel probabilistic discriminations involving different pairs of visual stimuli (Fig. 1, D5-8). Progression onto the next discrimination occurred after attaining criterion of 90% correct performance. The first 2 discriminations were presented with (D5) and without (D6) aversive loud noise punishment respectively, at 0.8 probability. The third and fourth discriminations were with punishment and a probability of 0.7 (D7) and 0.8 (D8), respectively.
3. two probabilistic discrimination reversals. Here, the contingencies were reversed such that the stimulus that was rewarded 80% of the time and punished 20% of the time was now punished 80% of the time and rewarded 20% of the time and vice versa for the other stimulus (Fig 1, D8 Rev1). After attaining 90% criterion on reversal 1, the probabilistic reward and punishment contingency reversed again (Fig 1, D8 Rev2).

Of the 12 animals in this study, 9 of them (3 per group) received an additional series of reversals immediately after the PVDLR task varying in 1) whether punishment was present or not and 2) whether the previously rewarded stimulus or the previously punished stimulus was replaced with a novel stimulus. Performance on these additional reversals was highly variable, even within the control group, where no consistent pattern of performance across different reversal types could be determined, and so these additional data are not reported here. As a consequence, the remaining 3 animals (1 per group) did not receive these additional reversals and went on to receive the VI test of punishment sensitivity immediately after completion of the PVDLR task and microdialysis. See Table 1 for study design details.

**Table 1:** Study design details

		PVDLR training		PVDLR testing	Additional reversals	Dialysis (months after surgery)	VI punishment sensitivity	Postmortem analysis (months after surgery)
Control	1	✓	Surgery	✓	✓	16.1	✓	17.4
	2	✓		✓	✓	14.7	✓	15.7
	3	✓		✓	✓	15.9	✓	21.9
	4	✓		✓	–	3.4	✓	9.0
Amygdala	1	✓		✓	✓	15.1	✓	17.0
	2	✓		✓	✓	14.0	✓	15.2
	3	✓		✓	✓	16.6	✓	17.0
	4	✓		✓	–	4.5^a^	–	5.0
OFC	1	✓		✓	✓	17.6	✓	19.2
	2	✓		✓	✓	15.6	✓	15.7
	3	✓		✓	✓	13.5	✓	15.0
	4	✓		✓	–	4.7	✓	9.9

^a^Animal never fully recovered from microdialysis and was euthanized.

#### VI Test of Punishment Sensitivity

Having completed probabilistic discriminations and reversals and received microdialysis, the animals were trained on a VI schedule of reward. In the first stage of training, the animals had to respond to a circle (radius = 3 cm) displayed on one side of the touch screen and associated with reward delivery on a VI schedule (5–15 s in 5-s steps). Thus, after the VI had elapsed, the next response to the stimulus resulted in the delivery of milkshake to the licker. In each session, the circle was displayed, first on one side of the touch screen and then on the other side, for each of 5 min. In the next stage, the animals had to respond to 2 circles displayed simultaneously on both sides of the touch screen with independent reward schedules. When marmosets were responding equally to both sides and getting nearly all the rewards, the animals proceeded to the tests of reward and punishment sensitivity.

Each animal received 10, 10-min sessions, the first 5 in the absence, and the second 5 in the presence of response-contingent mildly aversive loud noise. Initially, responding on both the right and left stimuli resulted in reward (duration, 6 s) delivered on independent 20-s VI schedules (0–40 s in 5 s steps). Thus, the first response, after 0, 5, 10, 15, … , 40 s had elapsed, resulted in reward. The next interval was not initiated until a response had been made after the end of the previous interval. In the second stage, 40-s VI punishment schedules (20–60 s in 5 s steps) with constant and equal frequency of aversive loud noise (0.3-s duration, 108 dB) was superimposed over the reward schedule on both left and right stimuli. Thus, the first response after 20, 25, 30, 35, … , 60 s had elapsed, resulted in punishment. These schedules were both independent of the reward schedules and each other. No single response was rewarded and punished. If an interval for both the punishment and reward schedules on a particular side had elapsed before an animal made the next response, then that next response was associated with the outcome from whichever schedule had elapsed first. The outcome from the other schedule was delivered contingent on the subsequent response.

### Behavioral Measures

#### Probabilistic Visual Discrimination

The main measure of monkeys' performance was the total number of errors made prior to achieving criterion of >90% correct responses (excluding the day on which the criterion was attained) on each discrimination. Additionally, Win–Stay/Lose–Shift (WSLS) behavior was analyzed according to the outcome of each preceding trial to assess the sensitivity of animals to positive and negative feedback. The veracity of the reinforcement was also taken into account, for example, whether the reinforcement was “true” (majority, e.g., reward following selection of the “correct” stimulus or punishment following selection of the “incorrect” stimulus), or “false”/misleading (minority, e.g., punishment following selection of the correct stimulus and reward following selection of the “incorrect” stimulus).

#### VI Test of Punishment Sensitivity

The main measure of punishment sensitivity was the total number of responses made to each of the 2 stimuli presented on the left and right sides of the touch screen per session during the presence of punishment compared with its absence. Other measures included the latency to 1) initiate responding to the touch screen after receiving reward or punishment (initiation latency) and 2) make a response to the touch screen after a time interval has elapsed (completion latency) and thereby receive punishment or gain access to reward, by licking at the spout.

### Computational Modeling of Behavior

Eight reinforcement learning models, standard in the reinforcement learning literature, were fitted to the behavioral data and compared. Characteristics of all the models are described in detail in Supplementary Methods. Models were initially fitted by a maximum a posteriori method and compared using the Bayesian information criterion, with calculation in addition of the corrected Akaike information criterion.

The characteristics of the models were as follows:

When 2 stimuli were offered, the probability of choosing stimulus *i* was determined by the value assigned to each, *x_i_*, via the softmax function with unitary inverse temperature *β* = 1:

$$P(\text{actio}{\text{n}}_{i})={\text{softmax}}_{\beta }^{i}(x_{1}...\;x_{n})=\frac{e^{\beta x_{i}}}{\sum_{k=1}^{n}e^{\beta x_{k}}}$$

The value of *x* for each stimulus in the pair was determined as a function of reinforcement and/or measures of side “stickiness” l and stimulus stickiness c (described next):

$$x_{i,t}=v_{i,t}+l_{i,t}+c_{i,t}$$

Not all models used all parameters. Model parameters included: 1) sensitivity to reinforcement with (*τ*_r_ and *τ*_p_, rates) or without (*τ*_rp_) different response rates to reward and punishment; 2) “stimulus stickiness,” the tendency to repeat choices to stimuli that have been recently chosen (*τ_c_*, rate; *d_c_*, maximum effect relative to reinforcement); and 3) “side stickiness,” the tendency to repeat choices to the side (left vs. right) that had been recently chosen (*τ*_lc_, rate; *d*_lc_, maximum effect relative to reinforcement). All combinations of the following settings were tested: 1) *τ* versus *τ*_r_ and *τ*_p_; 2) stimulus stickiness used or not; 3) side stickiness used or not. Where a mechanism was not used, its contribution to *x_i,t_* was set to zero. The measures were calculated as described in Supplementary Methods.

#### Parameter Estimation

Parameters were estimated by hierarchical Bayesian inference using Stan [Stan Development Team. 2013. Stan: A C++ Library for Probability and Sampling, Version 1.3, http://mc-stan.org/]. For each parameter, a value was drawn for each subject from a normal distribution. That normal distribution had a parameter-specific and group-specific mean, and a parameter-specific but group-shared standard deviation. Group mean rates (for *τ, τ*_r_, *τ*_p_, *τ_c_*, *τ_l_*) were drawn from a Beta(1.1, 1.1) prior distribution, and rates were constrained to the range [0,1]. Group mean maxima (for *d_c_*, *d_l_*) were drawn from a Gamma(shape = 1.2, rate = 0.2 [scale = 5]) prior distribution, and maxima were constrained to be positive. Standard deviations, for all parameters, were drawn from a positive-half-Cauchy(0, 5) prior distribution (and, of course, were constrained to be positive). Values of primary interest that were sampled were the posterior distributions of the group means, and the posterior distribution of differences between pairs of group means. Highest density intervals (HDI) were constructed; for example, a 95% HDI is the narrowest interval that contains 95% of the sampled values, and is not necessarily symmetric about the mean. Note that HDIs convey information about posterior probabilities and relate to the believability of specific parameter values; in this case, *P*(parameters | data, priors, model), unlike frequentist *P*-values or confidence intervals, which convey information about *P*(data | null hypothesis) and do not directly convey information about the believability of parameter values (Kruschke 2011).

#### Model Comparison

The log-likelihood, Σ_trials_ ln(*P*(actual choice | parameterized model)) was also sampled, yielding the mean maximized posterior log-likelihood LL. The Bayesian Information Criterion (BIC) was calculated for each model as –2 LL + *k* ln(*n*), where *n* was the total number of trials and *k* = *zs*, where *s* is the number of subjects and *z* is the number of parameters per subject entering the reinforcement learning model. The model with the lowest BIC value was selected as the best; that is, the model giving the highest log-likelihood, having penalized for model complexity. [Note that the original BIC was expressed as BIC = LL – 0.5 *k* ln(*n*), for which the largest is the best (Schwartz 1978; Burnham and Anderson 2004).]

#### Model Predictions for Behavioral Performance

To compare the model's predictions to the WSLS analysis taking into account the veracity of the feedback, the probabilities of “obeying” true or false (misleading) feedback were sampled from the best-fit computational model of behavior. Per-subject estimates were sampled of the mean probability (as determined by the model) of choosing an option that would correspond to obeying, given the actual choice made and actual reinforcement obtained on the previous trial.

In a further analysis, we examined which parameters in the best-fit model were capable of generating the effects seen in the statistical analysis of behavior. For each lesion group, an arbitrary high number of virtual subjects was run (*n* = 1000). Each virtual subject within a given group took the same parameter values, and performed in a virtual task, with a valid-reinforcement probability of 0.8, 30 trials per session, and a stopping criterion of 90% correct within a session. Simulated choices were drawn based on the reinforcement learning model's probability of selecting each stimulus at that moment, and then overall behavioral measures (errors to criterion, probability of obeying true and false feedback) were compared across virtual groups with a *t*-test (having arbitrarily high power, and thus useful only for detecting if parameter changes were in principle capable of producing an observed behavioral effect). For each set of comparisons, a given parameter was either given a group-specific value (e.g., differing between control, OFC, and amygdala virtual groups, with the actual values being the appropriate group mean best-fit parameter value) or constrained to be the same across groups (the mean best-fit parameter across all subjects). As an example, one comparison allowed the reinforcement rate parameter to vary across groups but the other 4 parameters were the same across groups; this tests whether variation in the reinforcement rate parameter was sufficient to produce the observed behavioral effects. All possible sets of these comparisons were run, allowing necessity as well as sufficiency to be tested (see Supplementary Material).

### Surgical Procedure

Selective depletions of 5-HT in the OFC and amygdala were made using 5,7-DHT (9.92 mM; Fluka BioChemika, Sigma, Poole, United Kingdom) in saline/0.1% l-ascorbic acid. To protect noradrenaline (NA) and dopamine (DA) innervations, the solution also contained the NA uptake blocker, nisoxetine hydrochloride (50 mM; Sigma), and DA uptake blocker, GBR-12909 dihydrochloride (2.0 mM; Sigma). Injections were made at a rate of 0.05 μL/20 s through a glass cannula attached to a 2-μL Hamilton syringe (Precision Sampling Co., Baton Rouge, LA, USA). The coordinates and volumes of toxin administered are shown in Table 2. Sham-operated controls (2 amygdala and 2 OFC) underwent identical surgical procedures with the toxin omitted from the infusate. Details of the surgical procedure are described in Supplementary Methods.

**Table 2:** Stereotaxic coordinates for 5-HT depletions

Depletion area	Coordinates (mm)			Volume injected (μL)
	AP	LM	V	
OFC	16.75	±2.5	0.7^a^	0.4
		±3.5	0.7^a^	0.4
	17.75	±2.0	0.7^a^	0.4
		±3.0	0.7^a^	0.4
	18.50	±2.0	0.7^a^	0.6
AMYGDALA	9.30	±5.6	4.0	0.5
			5.0	0.5

Coordinates are based on the interaural plane except for ^a^where the vertical was 0.7 mm above the base of the brain. AP, anterior–posterior; LM, lateral-medial; V, ventral.

### In Vivo Assessment of Extracellular 5-HT Using Microdialysis

Given the duration of the study and the finding in previous studies that 5,7-DHT-induced depletions of 5-HT, especially in the amygdala, show considerable recovery over time, the extracellular levels of 5-HT were assessed using in vivo microdialysis in anesthetized animals, after they had completed the probabilistic discriminations but before they were tested on the VI test of punishment sensitivity (mean: 12.6 ± 1.5, range: 3–18 months after surgery; see Table 1 for individual time points). This allowed us to obtain an additional measure of the extent of the depletion at a time point when the animals had just completed the probabilistic discrimination test. Following isoflurane anesthesia, probes were inserted acutely in the following stereotaxic coordinates based on the interaural plane: amygdala: AP +9.0 mm, L −5.6 mm, DV +4.0 mm; OFC: AP +17.25 mm, L +2.5 mm, DV +0.7 mm from the base of the skull. Sham-operated control animals were dialyzed on 2 separate occasions, at least 3 weeks apart, in order to measure extracellular levels of 5-HT in both the amygdala and OFC. OFC and amygdala 5-HT-depleted animals were dialyzed in the OFC and amygdala, respectively. Details of the microdialysis procedure are described in Supplementary methods.

### Neurochemical Assessment of Postmortem Brain Tissue

Postmortem neurochemical analysis was used to determine the specificity and extent of the 5,7-DHT depletion in the OFC and amygdala groups. On completion of the study (mean: 14.8 ± 1.3, range: 5–22 months after surgery; see Table 1 for individual time points), animals were euthanized with Dolethal (1 mL of pentobarbital sodium, 200 mg/mL solution i.p.; Merial Animal Health, Ltd, Essex, United Kingdom), and tissue samples were taken from cortical and subcortical regions. Samples were homogenized in 200 μL of 0.2 M perchloric acid, centrifuged at 6000 rpm for 20 min at 4 °C, and the supernatant was analyzed by reversed-phase HPLC and electrochemical detection, described in detail in Supplementary Methods.

### Statistics

Data were analyzed using SPSS for Windows (version 16.0, SPSS, Inc., Chicago, IL, USA). Statistical significance was set at *P* < 0.05 and where variances deviated significantly (as measured by Levene's test of homogeneity), data were transformed so that the variances were no longer significantly different. The nature of any transformation is indicated in the relevant section of the results. Neurochemical data were calculated as percentage change from control and analyzed using one-sample *t*-tests. For the probabilistic discrimination task, the numbers of errors to criterion for each of the 4 discriminations in the acquisition phase and the 2 reversals in the reversal phase were compared across the 3 groups with a two-way ANOVA. Additional two-way ANOVAs compared errors to criterion across the different discriminations and reversals of each lesion group with the control group. Subsequently, WSLS data were analyzed using a four-way ANOVA with within-subject factors of WSLS_Win-Stay/Lose-Shift_, Veracity_True/False_, and Phase_Acquisition/Reversal_ and between-subject factor of Lesion group_Control/AMYG/OFC_. The punishment sensitivity data were analyzed using two-way ANOVA with a within-subject factor of Punishment_Reward/RewardAndPunishment_ and a between-subject factor of Lesion group. Additionally, the difference in numbers of responses made by the experimental groups, before and after punishment introduction, was analyzed using one-way ANOVA. The latency data were analyzed using two-way ANOVA with within-subject factor of Trial_Rewarded/Punished_ and between-subject factor of Lesion group. Planned comparisons were carried out using simple main effects using the mean square error term from the original interaction (Winer 1971). Group comparisons were made using Fisher's protected least significant difference (LSD) tests. All data are presented as mean ± standard error of the mean (SEM). For all ANOVAs, type III sums of squares were used.

## Results

### 5,7-DHT Infusions into the OFC and Amygdala Reduce Postmortem Tissue Levels and In Vivo Extracellular Levels of Brain 5-HT

**Figure 2.**
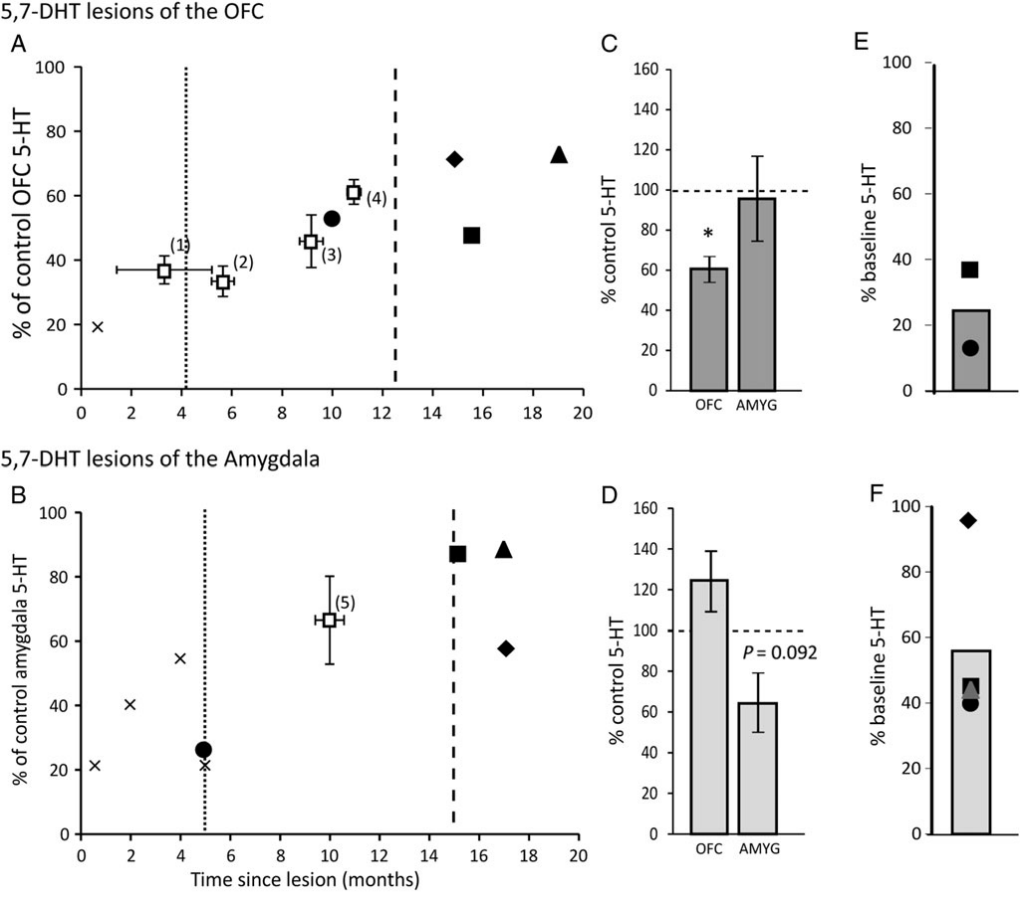
Reductions in ex vivo tissue levels and in vivo extracellular levels of brain 5-HT following 5,7-DHT infusions into the OFC and amygdala. (*A* and *B*) 5-HT levels in the OFC of 5,7-DHT OFC-infused animals (*N* = 4) and the amygdala of 5,7-DHT amygdala-infused animals (*N* = 4), respectively, as a percentage of controls (*N* = 4), at various time points, after surgery. The dotted and dashed lines indicate the mean number of months, after surgery, that 5-HT-depleted monkeys completed the probabilistic discrimination and punishment sensitivity tests, respectively. The filled symbols represent individual 5-HT-depleted animals in the present study, while the crosses represent animals that had received unilateral infusions (previously reported in the case of the amygdala: (Man et al. 2014)). The open squares represent data from bilaterally infused animals from previously reported experimental studies^(1–5)^. (*C* and *D*) Mean levels of postmortem tissue levels of 5-HT in the OFC and amygdala of 5,7-DHT OFC-infused and 5,7-DHT amygdala-infused animals, respectively, expressed as a percentage of controls. (*E* and *F*) Mean and individual percentage change from control levels of in vivo OFC extracellular 5-HT in 5,7-DHT OFC-infused animals and in vivo amygdala extracellular 5-HT in 5,7-DHT amygdala-infused animals, respectively. Where levels fell below the limit of detection of 7.5 fmol, percentage decreases were calculated using this detection limit (circle in *E* and circle and triangle in *F*). ^1^Four 5,7-DHT OFC-depleted marmosets previously reported in (Clarke et al. 2007), study 1: ^2^Four 5,7-DHT OFC-depleted marmosets previously reported in (Walker et al. 2006), ^3^Four 5,7-DHT OFC-depleted marmosets previously reported in (Walker et al. 2009), ^4^Eight 5,7-DHT OFC-depleted marmosets previously reported in (Clarke et al. 2007), study 2: ^5^Four 5,7-DHT amygdala-lesioned marmosets previously reported in (Man et al. 2014). See also Supplementary Table 1.

Injections of 5,7-DHT into the OFC induced a significant (*t*_3_ = −8.10, *P* = 0.004) reduction of 5-HT in the OFC (compared with sham-operated controls), as measured in postmortem tissue, 10–19 months after surgery (Fig. 2*A,C*). This reduction was regionally selective with no significant alterations in neighboring prefrontal or cingulate regions (largest *t*_3_ = 2.86, ns; Supplementary Table 1A). It was also neurochemically selective with no corresponding reductions of DA or NA within the OFC (*t*s ≤ 1) or surrounding tissue (largest *t*_3_ = −1.6, ns; Supplementary Table 1B). Although the mean level of depletion by the end of the study was only 38.98%, that is, levels that were 61.02 ± 6.36% of controls (Fig. 2*C*), findings from previously published experimental studies from our laboratory reveal much greater depletions at earlier time points, when the majority of the behavioral studies occurred (Fig. 2*A*, see dotted and dashed lines for mean time points of behavioral testing). Moreover, in vivo microdialysis in 2 of the 4 animals, immediately after the PVDLR task, and just before the VI test of punishment sensitivity, confirmed that extracellular levels of 5-HT within the OFC showed substantial reductions (Fig. 2*E*), demonstrating that 5-HT activity in the OFC was still markedly compromised. The samples from the remaining 2 animals could not be analyzed as they were inadvertently thawed when a freezer failed.

A similar mean level of depletion was seen following 5,7-DHT injections into the amygdala. However, due to increased variability in the amygdala group, compared with the OFC group, percentage change from controls, measured 5–17 months after surgery, was not significant (*t*_3_ = −2.46, *P* = 0.092; Fig. 2*D*), although 2 of the 4 animals had levels that were 57.4% (diamond) and 26.5% (circle) of control mean levels (Fig. 2*B*). Moreover, in vivo microdialysis revealed that 3 of the 4 monkeys (Fig. 2*F*) had marked reductions of extracellular 5-HT (<42%, <41%, and 45.1% of control). Data from our previously published experimental studies have demonstrated that reductions of 5-HT during the first 6 months after surgery (when the majority of behavioral testing took place) are on average, 40% of control (Fig. 2*B*). Consistent with this, in the one amygdala-lesioned animal in which brain tissue was analyzed at just 5 months post surgery, levels were 26.5% of control (Fig. 2*B*). Levels of DA and NA were unaffected (*t*s < 1; Supplementary Table 1B).

### 5-HT Depletion in the Amygdala or OFC Impairs Acquisition and Reversal of Probabilistic Discriminations

**Figure 3.**
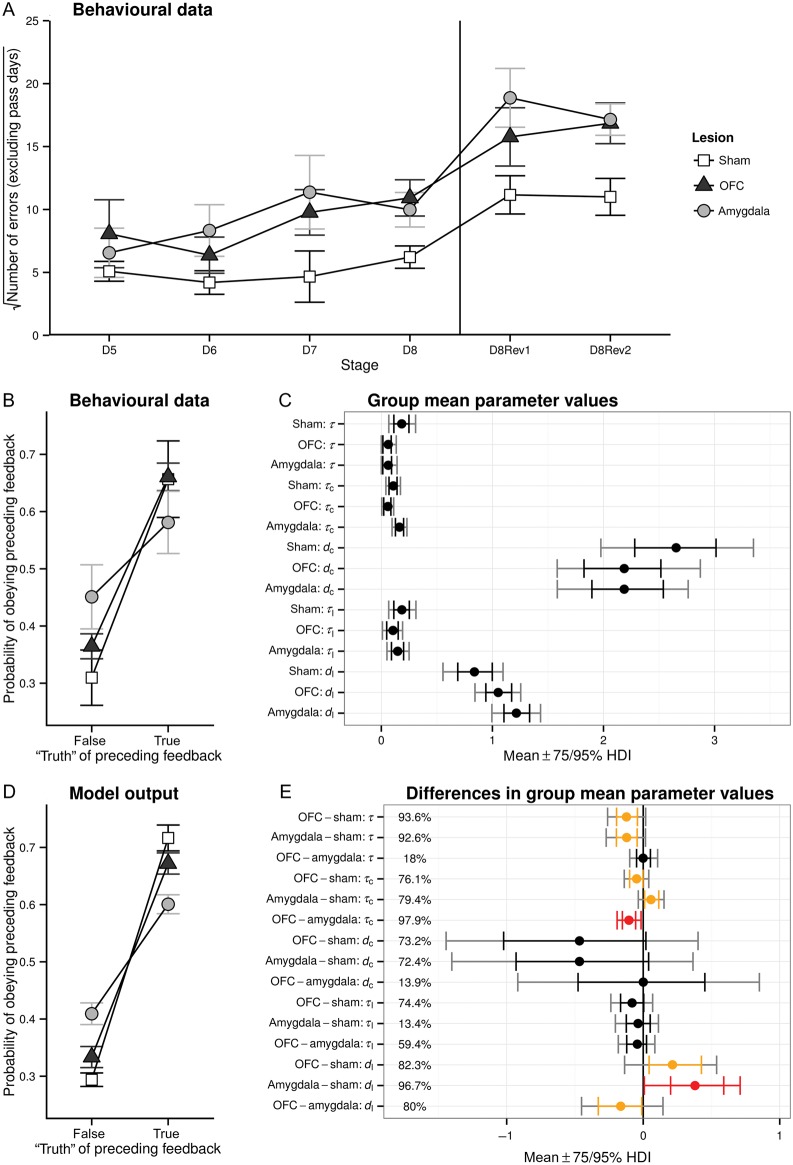
Lowered 5-HT in the amygdala or OFC impaired probabilistic discrimination and reversal. (*A*) Number of errors prior to reaching criterion performance (excluding the day on which the criterion was passed), square-root transformed, for each stage. (*B*) The probability of “obeying” feedback from the preceding trial (i.e., of staying with the previously chosen stimulus following reward, or switching to the alternative stimulus following punishment). Data are from discrimination learning stages (D5–D8) only. The response to “true” (majority) or “false” (minority) feedback is shown separately; the proportion of majority feedback trials was 80% (D5, D6, D8) or 70% (D7). (*C*) Group mean parameter values from the best-fit computational model of behavior (see text). Points indicate the mean of the posterior distribution of each parameter; error bars are 75% and 95% highest density intervals (HDIs). The computational model was fitted using data from discriminations (D5–D8) only. (*D*) The probability of “obeying” feedback, as for (B), but calculated using per-subject mean probabilities of “obeying” each kind of feedback, sampled from the best-fit computational model of behavior. The similarity to (B) indicates that the model successfully captured these aspects of behavior, even though the model incorporated no information about the “veracity” of preceding feedback. (*E*) Group mean differences in parameters from the best-fit computational model of behavior (see text), shown as the mean of the posterior distribution of each comparison parameter, directly sampled using Monte Carlo techniques from the hierarchical Bayesian inference model. Error bars are HDIs as before (orange, 75% HDI excludes zero; red, 95% HDI excludes zero). Percentages are the posterior probabilities that the parameter differs from 0 (width of the largest HDI excluding zero). The computational model was the same as that in (*C*).

Animals destined to receive either selective 5-HT depletions within the amygdala, OFC, or control surgery did not differ in their ability to learn a series of visual discriminations (one-way ANOVA errors to criterion, *F* < 1). Nor did they differ in their ability to remember a previously learned visual discrimination (one-way ANOVA errors to criterion, *F* < 1) postoperatively (mean errors to regain criterion, control 0.61 ± 0.6, 5-HT AMYG 1.8 ± 1.1, 5-HT OFC 0.6 ± 0.7). However, 5-HT depletions in either locus did significantly impair the acquisition and reversal of a series of novel discriminations, as assessed by the number of errors made before attaining criterion performance (Fig. 3*A*). The depleted groups did not differ from each other, and there was no interaction with discrimination/reversal stage. There were no differences between individual discriminations (despite some differences in reinforcement probability and the aversive outcome) or between individual reversals; unsurprisingly, more errors were made during reversals than during discrimination learning.

The number of errors made by each subject on each stage was square-root transformed (because their variance increased with the mean) and then analyzed by ANOVA. In the first analysis, the factors were Lesion (control, OFC, amygdala) and Stage (D5, D6, D7, D8, D8Rev1, and D8Rev2). There was a main effect of Lesion (*F*_2,9_ = 10.0, *P* = 0.005) and of Stage (*F*_5,45_ = 3.31, *P* = 0.012), with no Lesion × Stage interaction (*F* < 1). This effect was explored by considering lesion groups separately, using similar ANOVAs but with only two groups at a time. Comparing the OFC-lesioned and the control groups, there was similarly a Lesion effect (*F*_1,6_ = 9.73, *P* = 0.021), a Stage effect (*F*_5,30_ = 4.52, *P* = 0.003), and no interaction (*F* < 1). Comparing the amygdala and control groups, there was a Lesion effect (*F*_1,6_ = 47.0, *P* = 0.000), a Stage effect (*F*_5,30_ = 3.064, *P* = 0.024), and no interaction (*F* < 1). However, there were no differences between OFC and amygdala groups: although there was a Stage effect (*F*_5,30_ = 4.82, *P* = 0.002), there was no effect of Lesion and no interaction (*F*s < 1).

As illustrated in Figure 3*A*, the Stage effect was evidently contributed to by the difference between discriminations (D5–D8) and reversals (D8Rev1 and D8Rev2). To establish whether differences between individual discriminations or between individual reversals were important, the three-group analysis was repeated using only the discrimination stages, or only the reversal stages. For the discrimination stages, there was no effect of Stage and no Stage × Lesion interaction (*F*s < 1). Similarly, there was no effect of Stage within the reversal sessions and no interaction (*F*s < 1). Thus, there was no evidence of differences between individual discrimination stages, or between individual reversal stages.

### 5-HT Depletion in the Amygdala Increases Sensitivity to Misleading Feedback

The amygdala-depleted subjects were more likely than controls or OFC-depleted subjects to “obey” false/misleading (minority), but not true (majority) reinforcement. Thus, they were more likely to shift in response to false punishment and stay in response to false reward and overall, showed less discrimination between true and false reinforcement (Fig. 3*B*).

Every trial was matched with the preceding trial (within sessions) to establish whether the subject “obeyed” the preceding feedback (meaning that it stayed with the chosen stimulus on the second trial following reward on the first, or switched to the other stimulus following punishment). Obey probabilities were calculated for each subject across all valid trial pairs and analyzed with ANOVA. We expected that reward “veracity” would become less meaningful during reversal learning, since previously true feedback becomes false and the animal's expectations change over the course of a reversal learning session; however, we did not want to miss the possibility of lesion effects that differed between discrimination and reversal learning. Therefore, we first analyzed discrimination and reversal phases together; this indicated a robust lesion effect but no difference between the two phases. In this first analysis, the factors were Lesion (as before), SessionType (discrimination versus reversal), Valence (reward or punishment on the previous trial), and Veracity (whether the preceding trial's reinforcement had been true, meaning in the majority, or false, meaning in the minority and misleading as to the overall best stimulus). No terms involving SessionType were significant, although there was a Lesion × Veracity interaction (*F*_2,9_ = 16.5, *P* = 0.001). Therefore, we subsequently excluded the reversal learning phases and analyzed only the discrimination phase. In this reanalysis, the Lesion × Veracity interaction remained significant (*F*_2,9_ = 10.1, *P* = 0.005).

We analyzed this Lesion × Veracity interaction by considering the effects of Lesion for true and false feedback, respectively. For true feedback, there was no effect of Lesion (*F*_2,9_ = 2.87, *P* = 0.109). For false feedback, there was a highly significant effect of Lesion (*F*_2,9_ = 12.4, *P* = 0.003); pairwise comparisons between groups demonstrated that the amygdala-depleted groups were more likely to obey misleading feedback than controls (*F*_1,6_ = 26.1, *P* = 0.002) or OFC-depleted animals (*F*_1,6_ = 8.32, *P* = 0.028), while the OFC and control groups did not differ significantly (*F*_1,6_ = 3.76, *P* = 0.101).

### 5-HT Depletion in the Amygdala or OFC Reduces Reinforcement Sensitivity as Determined by Computational Modelling

Typically, WSLS analysis, as used above and in many previous studies (Murphy et al. 2003; Bari et al. 2010; den Ouden et al. 2013), only assesses sensitivity to immediately received feedback and fails to take account of the possible integration of reinforcement feedback across past trials. In order to determine the degree to which the 5-HT-depleted and control groups were influenced by immediately preceding reinforcement versus their cumulative past history of reinforcement, eight reinforcement learning models were fitted to the postoperative discrimination (D5-D8) data and compared (see Supplementary Table 1). The best model, as judged by the BIC, was one that incorporated parameters for 1) sensitivity to reinforcement (*τ*, rate), without the need for different response rates for reward and punishment; 2) stimulus stickiness, the tendency to repeat choices to stimuli that have been recently chosen (*τ_c_*, rate; *d_c_*, maximum effect relative to reinforcement); and 3) “side stickiness,” the tendency to repeat choices to the side (left vs. right) that had been recently chosen (*τ*_lc_, rate; *d*_lc_, maximum effect relative to reinforcement). This model was a good descriptor of postoperative performance in all groups and also won when all postoperative data, that is, discriminations and reversals, were included. Additionally, it won as judged by the corrected Akaike information criterion, AIC*_c_* = [2*k* − 2LL] + [2*k*(*k* + 1)/(*n*− *k*− 1)].

The winning model provided good evidence for reduced reinforcement sensitivity in marmosets with 5-HT depletions in either the amygdala or the OFC (lower *τ*; Fig. 3*C,E*), compared with controls (0.926 ≤ *P*_NZ_ ≤ 0.936; *P*_NZ_ denotes the probability that the posterior credible interval of the difference, specifically the HDI, excludes zero). In addition, there were also changes in the stickiness parameters. In particular, 5-HT depletion in the OFC and amygdala induced opposing effects on changes in stimulus stickiness (OFC *τ_c_* *<* amygdala *τ_c_*, *P*_NZ_ = 0.979). The OFC-depleted group displayed less rapid changes (compared with controls, lower *τ_c_*, *P*_NZ_ = 0.761), indicating that their tendency to repeat choices to recently chosen stimuli operated over a longer timescale. In contrast, the amygdala-depleted group displayed more rapid changes (compared with controls, higher *τ_c_*, *P*_NZ_ = 0.794), indicating that their tendency to repeat choices to recently chosen stimuli was more ephemeral. The overall effect of side stickiness (relative to that of reinforcement) was higher in both the OFC and the amygdala groups (*d_l_*, 0.823 ≤ *P*_NZ_ ≤ 0.967), somewhat more so in the amygdala group (OFC *d_l_* *<* amygdala *d_l_*, *P*_NZ_ = 0.800). There were no marked group differences (*P*_NZ_ < 0.75) in the maximum parameter governing stimulus stickiness (*d_c_*) or the rate parameter governing side stickiness (*τ_l_*).

To assess the validity of the model, we determined the pattern of behavioral performance it predicted in relation to errors to criterion and the WSLS analysis. It successfully retrodicted the observation of increased errors to criterion (model: average square-root-transformed errors per discrimination; control 3.78 < amygdala 4.83 < OFC 5.42) and even though this computational model did not explicitly represent the veracity of preceding feedback (false or true feedback), it also successfully reproduced the patterns of behavioral sensitivity to feedback (compare Fig. 3*D* with *B*), with similar changes in the tendency to obey false feedback. In addition, to establish the necessity and sufficiency of the changes in the model parameters to account for these behavioral differences (i.e., changes in errors to criterion and changes in responsivity to misleading feedback), multiple (*n* = 1,000) virtual subjects per group were simulated, using the best model, but allowing only individual or subsets of the parameters to vary between the groups (see Supplementary Results for details). This revealed that between-group variation in the reinforcement rate was necessary and sufficient to explain these behavioral differences in the amygdala-depleted group, with changes in stimulus and side stickiness alone, or in combination, failing to explain such differences. Reinforcement rate differences were also sufficient to explain them in the OFC-depleted group but, in this case, so too were differences in stimulus stickiness.

In summary, 5-HT depletion in both the amygdala and OFC impaired the learning of a series of probabilistic discriminations and reversals, and this impairment could be accounted for by reductions in the rate of reinforcement-driven learning across trials.

### 5-HT Depletion in the Amygdala or OFC Abolishes Punishment-Induced Response Suppression

**Figure 4.**
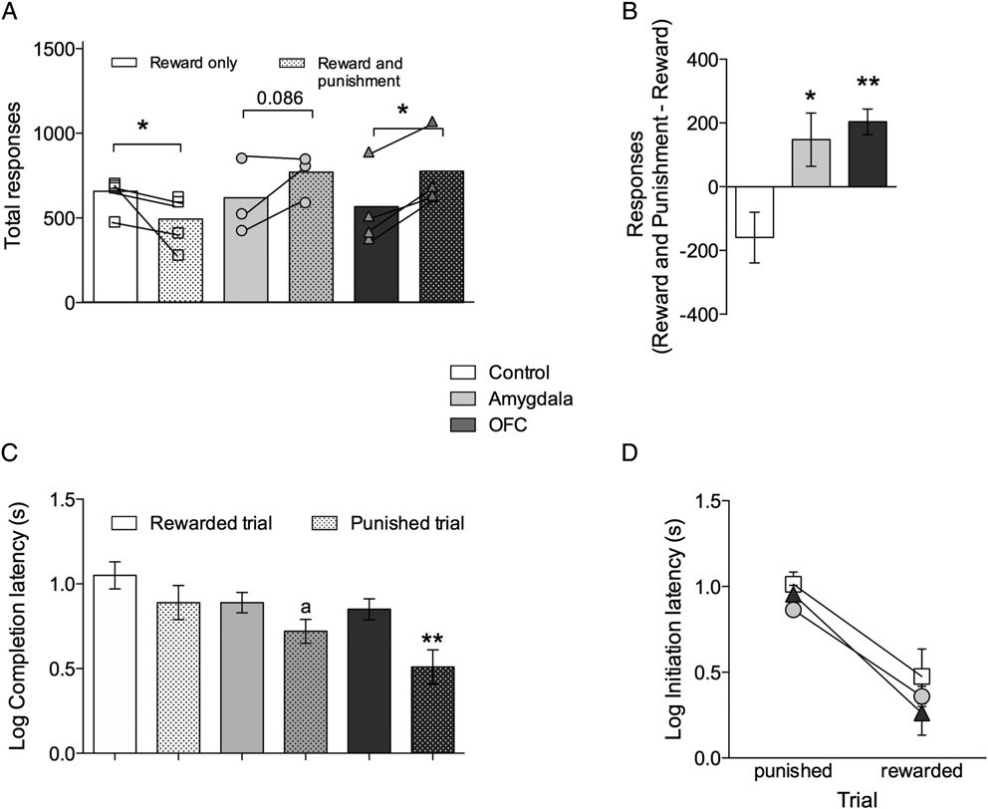
Depletion of 5-HT in the amygdala or OFC abolishes punishment-induced suppression but leaves intact sensitivity to aversive outcomes. (*A*) Total responses made by 5-HT amygdala- and OFC-depleted animals and sham-operated controls before (open bars) and after (hatched bars) the introduction of punishment. (*B*) Change in responding after punishment introduction. (*C*) Log latency to complete trials after the VI schedule has elapsed and (*D*) log latency to initiate responding after receiving punishment and reward. Data presented as mean ± SEM, *P ≤ 0.05, **P ≤ 0.01, ^a^*P* = 0.068.

Upon completion of the probabilistic discrimination and reversal stages and following in vivo microdialysis, the sensitivity of all animals to punishment was assessed. Using a VI schedule of reinforcement (20 s) that provided equal opportunities for reward delivery on both sides of the touch screen, experimental groups did not differ in their total number of responses for reward only (Fig. 4*A*). In contrast, upon introduction of the 40-s VI punishment schedule, with equal frequency of aversive loud noise (0.3-s duration, 108 dB) on both sides, the response suppression that occurred in the controls was not seen in either of the 5-HT-depleted groups (Fig. 4*A,B*). Indeed, both depleted groups increased their total number of responses during the punishment phase compared with the reward-only phase.

Two-way ANOVA with factors of Punishment and Lesion group revealed no significant effects of Punishment (*F* < 1) or Lesion group (*F* < 1) but a significant Punishment × Lesion group interaction (*F*_2.8_ = 8.703, *P* = 0.010). Simple main effects revealed significant (*P* = 0.041) response suppression after introduction of punishment in the control animals. In contrast, the 5-HT OFC-depleted animals showed significant (*P* = 0.014) and 5-HT-amygdala-depleted animals near-significant (*P* = 0.086) increases in the total number of responses made after punishment introduction.

Consistent with this overall increase in responding following the introduction of punishment, both 5-HT-depleted groups exhibited faster completion latencies on both reward and punishment trials (Fig. 4*C*). In other words, their general increase in responding after the introduction of punishment meant that they were quicker to make a response after each VI had elapsed. Two-way ANOVA of log-transformed completion latencies with factors of Trial (rewarded/punished) and Lesion group revealed a significant effect of Trial (*F*_1,8_ = 21.145, *P* = 0.002), Lesion group (*F*_2.8_ = 5.813, *P* = 0.028) and significant Trial × Lesion group interaction (F_2.8_ = 5.376, *P* = 0.033). Post hoc LSD tests revealed that both 5-HT-depleted groups were faster to complete a VI schedule compared with control animals (*P* = 0.001 and *P* = 0.048 for OFC and amygdala, respectively), particularly on punished trials (*P* = 0.004 and *P* = 0.068 for control vs. OFC and control vs. amygdala, respectively).

### 5-HT Depletions in the Amygdala or the OFC do not Affect Sensitivity to Aversive Outcomes

In contrast to the abolition of response suppression in anticipation of punishment, sensitivity to the receipt of punishment was equivalent across all three groups; all animals being slower to initiate a new trial immediately after receiving punishment, compared with reward (Fig. 4*D*). Two-way ANOVA of log transformed initiation latencies with a within-subjects factor of Trial (rewarded × punished) and between-subjects factor of Lesion group revealed a significant effect of Trial (*F*_1,8_ = 21.145, *P* = 0.002) but no significant effects of Lesion group (*F* < 1) or Trial × Lesion group interaction (*F* < 1). Post hoc LSD tests revealed that all experimental groups showed significant (*P* = 0.001, 0.001, and 0.002 for control, OFC, and amygdala groups, respectively) increases in their response initiation latencies for the next trial, having just receiving punishment, compared with reward.

## Discussion

The present study demonstrates that an apparent enhanced sensitivity to misleading punishing feedback and reduced punishment-induced response suppression are both present following localized 5,7-DHT-induced depletions of 5-HT in the amygdala of nonhuman primates. Specifically, reductions of 5-HT in either the amygdala or OFC disrupted probabilistic discrimination and reversal learning, with an increased effectiveness of misleading, or false, punishment and reward in the amygdala-depleted group, measured by WSLS analysis. 5-HT depletions in either the OFC or the amygdala led to an abolition of suppression of responding on a VI response task, indicative of a decrease in punishment sensitivity and/or a loss of behavioral control. Previously, such contrasting effects of 5-HT depletion have only been reported following global 5-HT manipulations in rodents and humans. Here, we show that they are still present following localized 5-HT manipulations in the brain in a study that has ruled out any explanations in terms of differences in the type of negative feedback, the experimental procedure used to deplete 5-HT, or dissociable functional effects in the amygdala and OFC. Computational modeling revealed that the apparent increased sensitivity to misleading feedback in the probabilistic discrimination task could be accounted for in terms of an overall reduction in reinforcement sensitivity, irrespective of affective valence. We propose that such an explanation can provide a unifying account of these apparently opposing behavioral effects.

### Methodological Considerations

5,7-DHT-induced depletions of 5-HT in the OFC and amygdala of marmosets show recovery over time, with maximum depletions of ∼80%, 1 month after surgery, declining to ∼35% in the OFC and <25% in the amygdala between 15 and 19 months after surgery. Given that 5-HT depletion in the amygdala recovers more quickly than that in the OFC, the most likely explanation for the larger variability in the post-mortem tissue levels of the amygdala group is the difference in the postoperative time point at which post-mortem tissue measurements were made (5–17 months) in the amygdala group. Thus, it is important to take into account the time course of recovery when interpreting the behavioral findings. Deficits in performance of the PVDLR task occurred within the first 5 months of testing when depletions are substantial (see Fig. 2*A*,*B*). In contrast, the failure to show punishment-induced response suppression in six of the seven 5-HT-depleted monkeys occurred at a much later time point in the majority of cases (between 13.5 and 18 months after surgery; Table 1), when depletion would have been much less. However, evidence that 5-HT neurotransmission was still compromised at this time point was shown by the reduced levels of extracellular 5-HT, as measured by in vivo microdialysis (Fig. 2*E*,*F*). Moreover, a similar failure in punishment-induced response suppression was also seen in the one OFC-depleted animal that was tested much earlier, at just 5 months after surgery. Thus, the deficits observed are all associated with compromised 5-HT neurotransmission within the OFC and amygdala. However, it will be important to determine in the future whether the behavioral effects that follow long-term changes in 5-HT levels induced by 5,7-DHT infusions could stem from adaptive changes in the function of specific 5-HT receptor subtypes, similar to the changes observed in other brain regions after prolonged alteration in 5-HT signaling (Cahir et al. 2007; Jennings et al. 2008).

### Increased Sensitivity to Positive and Negative Misleading Feedback on the Probabilistic Discrimination Task

Previous investigations into the effects of reducing or increasing 5-HT levels on the sensitivity to positive and negative feedback in probabilistic discrimination tasks differ from the present study in that there was often a greater emphasis on reversal learning, and they focused exclusively on misleading (false) negative feedback. In those studies, an acute low dose of citalopram, which most likely reduces 5-HT function (for review, see (Artigas et al. 1996)), disrupted discrimination/reversal performance in both humans (Chamberlain et al. 2006) and rats (Bari et al. 2010), and enhanced sensitivity to negative feedback. In contrast, an acute high dose of citalopram, which most likely increases 5-HT function, had the opposite effect, acting to reduce negative feedback sensitivity (Bari et al. 2010). While acute tryptophan depletion (ATD) in humans was without effect, other than to increase overall response latencies (Murphy et al. 2002), permanent depletions of 5-HT in rats induced by intracerebroventricular 5,7-DHT infusion disrupted performance and caused a temporary increase in sensitivity to misleading negative feedback and a prolonged decrease in sensitivity to a rewarded “correct” choice, that is, true positive feedback (Bari et al. 2010). Together, these findings implicate 5-HT in responsivity to both positive and negative feedback, in part, dependent on whether the manipulation is mild and acute, or severe and permanent. However, the site of action of 5-HT in these studies is unclear, although human functional imaging studies have implicated the dorsomedial prefrontal cortex (PFC) as a site for the proposed involvement of 5-HT in increases or decreases in sensitivity to negative feedback (Evers et al. 2005; Macoveanu et al. 2013). In contrast, both the amygdala and the PFC have been implicated in mediating the deleterious effects of misleading negative feedback in a functional imaging study of probabilistic reversal in depression (Taylor Tavares et al. 2008).

By investigating the effects of localized depletions of 5-HT in specific brain regions, the present study has been able to identify the brain regions within which 5-HT is acting to induce such effects. In contrast to the above experiments in rats and humans, the present study presented a series of novel probabilistic discriminations, prior to any reversals, and analyses focused on both misleading negative and misleading positive feedback. The finding that depletions in either region disrupted overall performance identifies the important role of 5-HT in both the OFC and the amygdala in acquiring and reversing probabilistic discriminations. Detailed analysis of the responsivity of the animals to positive and negative feedback revealed that 5-HT depletions of the amygdala, and to a far lesser extent, the OFC (trend level only), made animals more likely to stay after misleading reward and more likely to shift after misleading punishment. Since previous studies (Chamberlain et al. 2006; Bari et al. 2010) did not report the effects of 5-HT manipulations on misleading reward (only on misleading punishment), it cannot be ascertained whether the effects seen in the present study differ from those previous reports that implicated 5-HT selectively in misleading punishment.

By applying computational reinforcement learning models to subjects' discrimination performance, the apparent enhanced sensitivity to misleading reinforcement described in the present study can be accounted for, somewhat counterintuitively, in terms of an overall reduction in reinforcement sensitivity. The instrumental behavior required in the probabilistic discrimination task was best described by a model in which learning was driven by reinforcement (according to a simple delta rule operating at the same rate for reward and punishment) as well as stimulus stickiness (the tendency to choose the stimulus chosen on recent trials) and side stickiness (the tendency to choose the side of the touch screen chosen on recent trials). 5-HT depletions in the amygdala and the OFC reduced the impact of reinforcement on behavior (regardless of affective valence) with much less of an effect on stimulus or side stickiness. Changes in reinforcement sensitivity were necessary and sufficient for explaining the observed behavioral effects in the amygdala group and sufficient for explaining them in the OFC group, though changes in stimulus stickiness parameters offered a potential alternative or additional explanation in the OFC group. Importantly, this model also retrodicted the behavioral outcomes, namely, that both amygdala and OFC 5-HT-depleted monkeys learned the discriminations more slowly and that the amygdala and, to a lesser extent, the OFC-depleted groups were more affected by misleading reinforcement, regardless of affective valence.

### Reduction of Punishment-Induced Response Suppression on the VI Response Task for Reward

Consistent with the interpretation that amygdala and OFC 5-HT-depleted animals showed reduced reinforcement sensitivity on a series of probabilistic discriminations, these same animals were also less sensitive to the introduction of punishment when responding on a VI schedule for reward. Punishment-induced response suppression has been shown to be dependent on the central, but not the basolateral nucleus of the amygdala (Killcross et al. 1997). It is disrupted by global (Robichaud and Sledge 1969; Graeff and Schoenfeld 1970; Tye et al. 1977; Soderpalm and Engel 1991) and amygdala-specific depletion (Sommer et al. 2001) of 5-HT in rodents. The capacity of aversive conditioned stimuli to cause behavioral inhibition is also attenuated by ATD in humans (Crockett et al. 2012). The present study shows that a similar loss of response suppression can be induced by a localized reduction of 5-HT in either the primate amygdala or OFC. Indeed, the 5-HT-depleted animals not only showed no decline in response rate, but instead displayed an increase in responding, suggesting that the aversive loud noise had a nonspecific arousing effect on responding for food reinforcement that was unmasked by the loss of punishment-induced response suppression. This effect on response rate was specific to the punishment condition, since all groups showed similar overall rates of responding for food reinforcement in the absence of punishment. It is similar to the enhancing effects of an aversive stimulus on food-reinforced operant responding reported in rats with septal lesions (Dickinson 1975) and the facilitative effects of punishment on extravert's reward seeking behavior (Nichols and Newman 1986). It highlights the multiple, competing effects that an aversive stimulus may have on behavior, including response suppression and nonspecific arousal, mediated by parallel, interacting circuits (Gray and Smith 1969) and suggests that serotonergic inputs into the OFC and amygdala are not involved in the nonspecific arousing effects of punished stimuli.

A role for 5-HT in reinforcement sensitivity could involve either “reporting” or “anticipating” reinforcement. A failure in reporting reinforcement, in this case, aversive loud noise, is inconsistent with the finding that 5-HT-depleted animals, like controls, were slower to initiate responding following the receipt of punishment compared with reward, an effect indicative of intact reporting of punishment. In contrast, a deficit in the anticipation of aversive outcomes would leave intact any slowing of responding upon the actual receipt of an aversive outcome but still result in a failure to suppress responding across a block of sessions in anticipation of prospective punishment. Our results are thus consistent with the idea of a loss of reinforcement anticipation in this context. Indeed, similar findings of a loss of punishment-induced response suppression, but intact sensitivity to punishing outcomes, has been reported in humans following global ATD (Crockett et al. 2009, 2012).

### Implications for Existing Theories of 5-HT Function

Serotonin has been implicated in several affective and behavioral control processes including behavioral inhibition, representing aversive values or prediction errors, behavioral flexibility, and delay discounting (Soubrie et al. 1986; Cools, Robinson et al. 2008). More recent attempts to integrate these theories have led to proposals suggesting that 5-HT operates at the intersection of aversion and inhibition, promoting response inhibition in the face of aversive predictions (Dayan and Huys 2009; Cools et al. 2010; Boureau and Dayan 2011). Indeed, it has been proposed that a reduction in aversive stimulus-induced response suppression may result in enhanced cognitive engagement with aversive stimuli and thus more accurate detection, encoding, and prediction of them (Dayan and Huys 2009).

While such theories could account for the enhanced prediction of punishment following tryptophan depletion in humans (Cools, Robinson et al. 2008), they do not account so readily for the pattern of effects observed following 5-HT depletion in the PFC and amygdala in the present study. Here, a reduction in stimulus-induced response suppression occurred alongside an overall impairment (and not an improvement) in predicting punishment and reward in a probabilistic discrimination task. Moreover, the apparent increased responsivity to misleading punishment was accompanied by an apparent increased responsivity to misleading reward, implicating 5-HT in the OFC and amygdala in reward as well as punishment processing. A role for 5-HT in reward processing is consistent with our previous findings in which 5-HT depletion in the amygdala altered the expression of Pavlovian appetitive conditioned attentional responses (Man et al. 2014), and 5-HT depletion in the OFC disrupted acquisition of responding with appetitive conditioned reinforcement but not extinction (Walker et al. 2009). The lack of effect on responding during extinction also argues against a role for 5-HT in reporting aversive outcomes that occur through omissions of reward. Additional supporting evidence includes the increase in 5-HT efflux in the medial PFC of rodents in anticipation of reward (Merali et al. 2004) and instrumental responding for immediate (Martin-Ruiz et al. 2001) and delayed reward (Winstanley et al. 2006), the finding that putative 5-HT neurons in the macaque dorsal raphé nucleus code reward value (Bromberg-Martin et al. 2010) and display tonic activity while awaiting reward (Kranz et al. 2010; Hayes and Greenshaw 2011; Miyazaki et al. 2011) and the finding that ATD impairs both behavioral and neural representations of reward outcome value (Seymour et al. 2012).

Thus, the most parsimonious explanation of the results is that dorsal raphé 5-HT may provide an anticipatory signal for both rewarding and punishing motivational outcomes, providing empirical support for the recent proposal of Miyazaki et al. (2011). However, rather than emphasizing the importance of 5-HT in coupling rewarding and punishing motivational signals with behavioral inhibition, the present results emphasize the role of 5-HT in the anticipatory signal, per se. Thus, the nature of the behavioral deficit may depend on whatever use is made of the anticipatory signal: for example to inhibit responding (conditioned suppression), to maintain a particular choice (ignoring misleading feedback, as in the probabilistic discrimination task), or to increase response vigor as a function of reward magnitude (Cools et al. 2005). Of course, this explanation does not rule out the possibility that 5-HT depletion within the OFC and amygdala may have more than one effect. Indeed, besides a failure to integrate outcomes across both positive and negative reinforcement history, our results showed that 5-HT OFC depletion specifically increased the timescale of the animals’ tendency to choose the same option (stimulus) again, regardless of the outcome, an effect that could also account for the animals' pattern of behavioral impairments. A similar effect has been reported previously in humans with acute dietary tryptophan depletion (Seymour et al. 2012) and would certainly be consistent with the perseverative responding on a deterministic discrimination task we have previously reported in 5-HT OFC-depleted marmosets (Clarke et al. 2004, 2005, 2007) and which has also been reported in rats with stress-induced OFC 5-HT depletion (Lapiz-Bluhm et al. 2009).

### Implications for Understanding of Mood and Anxiety Disorders

Serotonin is implicated in both the pathophysiology and treatment of mood and anxiety disorders. Since a core feature of these disorders is dysregulation within orbitofronto-amygdala circuitry (Phillips et al. 2003; Bishop 2007; Milad and Rauch 2007; Drevets et al. 2008; Clark et al. 2009), a neurobiological and psychological account of the role of 5-HT in these circuits will not only inform our understanding of these disorders but may also lead to more effective treatments. Particular relevance to the present study is that altered connectivity within prefronto-amygdala circuitry has been reported in depressed patients, specifically in response to negative feedback in the reversal of the probabilistic discrimination task (Taylor Tavares et al. 2008). The present findings demonstrate that reductions of 5-HT localized to either the OFC or the amygdala in a nonhuman primate decrease reinforcement sensitivity, but suggest that this impairment is not specific to negative feedback and thus does not induce a negative attentional bias, per se. Rather, it reduces the impact on behavior of anticipating rewarding and punishing outcomes, consistent with the overall blunting of affect that occurs in depression.

## Supplementary Material

Supplementary material can be found at: http://www.cercor.oxfordjournals.org/.

## Funding

This work was supported by a Wellcome Trust programme grant (grant number: 089589/Z/09/Z; to T.W.R., B.J. Everitt, A.C.R., J.D., and B.J. Sahakian), a Medical Research Council (MRC) Programme grant (grant number: G0901884; to A.C.R.), and a J. S. McDonnell Foundation grant (grant number: 220020155;Principal Investigators: E. Phelps, T.W.R.; co-investigators: J.E. LeDoux and A.C.R.). R.N.C. was supported by a Wellcome Trust postdoctoral fellowship. The study was conducted within the University of Cambridge Behavioural and Clinical Neuroscience Institute, supported by a joint award from the MRC and the Wellcome Trust. Funding to pay the open access publication charges for this article was provided by the Wellcome Trust.

## Supplementary Material

Supplementary Data
