# Supplementary material for: Role of Central Serotonin in Anticipation of Rewarding and Punishing Outcomes: Effects of Selective Amygdala or Orbitofrontal 5-HT Depletion
Source: Cereb Cortex. 2014 May 30;25(9):3064–76. doi: 10.1093/cercor/bhu102 (PMC4537445; doi:10.1093/cercor/bhu102)
Supplement: Supplementary Data [file supp_25_9_3064__index.html]

Role of Central Serotonin in Anticipation of Rewarding and Punishing Outcomes: Effects of Selective Amygdala or Orbitofrontal 5-HT Depletion — Role of Central Serotonin in Anticipation of Rewarding and Punishing Outcomes: Effects of Selective Amygdala or Orbitofrontal 5-HT Depletion — Supplementary Data 

# Role of Central Serotonin in Anticipation of Rewarding and Punishing Outcomes: Effects of Selective Amygdala or Orbitofrontal 5-HT Depletion

## Supplementary Data

Supplementary Data

**Files in this Data Supplement:**

- Supplementary Data - Docx file
