# Supplementary material for: Role of Central Serotonin in Anticipation of Rewarding and Punishing Outcomes: Effects of Selective Amygdala or Orbitofrontal 5-HT Depletion
Source: Cereb Cortex. 2014 May 30;25(9):3064–76. doi: 10.1093/cercor/bhu102 (PMC4537445; doi:10.1093/cercor/bhu102)
Supplement: Supplementary Data [file supp_bhu102_bhu102supp.docx]

**Supplementary Material**

**Role of central serotonin in anticipation of rewarding or punishing outcomes: Effects of selective amygdala or orbitofrontal 5-HT depletion**

*R. Rygula, H.F. Clarke, R.N. Cardinal, G.J. Cockcroft J, Xia, J.W. Dalley, T.W. Robbins, A.C. Roberts*

**Methods**

**Surgical procedures**

Marmosets were pre-medicated with ketamine hydrochloride (0.1 ml of a 100 mg/ml solution, i.m.; Pfizer, Kent, UK) and a prophylactic analgesic (Norocarp; 0.03 ml of 50 mg/ml carprofen, s.c.; Pfizer), before being intubated and maintained on isoflurane gas anaesthetic (flow rate: 3.0% isoflurane in 0.3 l/min O_2_ ; Novartis Animal Health UK, Herts, UK). Animals were then placed in a stereotaxic frame (David Kopf Instruments, Tujunga, CA) with incisor and zygoma bars specially adapted for the marmoset.

Dexamethasone phosphate (0.2ml i.m.; Fauling Pharmaceuticals plc, Warwick, UK) was administered on completion of surgery to prevent tissue inflammation. The analgesic Metacam (meloxicam, 0.1ml of a 1.5mg/ml oral suspension; Boehringer Ingelheim, Germany) was given every 24 hours for three days post-operatively for further pain relief. Animals were returned to their home cage and had access to water *ad libitum* and supplementary diet during a recovery period of at least 10 days.

**In vivo Assessment of Extracellular 5-HT Using Microdialysis**

Commercially available BASi brain microdialysis probes with a 2mm membrane, (BASI MD-2200, BR-2 Bioanalytical Systems, Inc., Indiana, USA) were used for collection of dialysate. Harvard microsyringe pumps with 2.5mL gastight syringes perfused artificial cerebrospinal fluid (aCSF) through the dialysis probe at a flow rate of 1.0 μL/min. The aCSF had the following composition (mM): NaCl 147, KCl 3.0, CaCl_2_ 1.3, MgCl_2_ 1.0, NaH_2_PO_4_ 0.2, Na_2_HPO_4_ 1.3. After approximately three hours to allow the inserted probes to equilibrate, dialysate fractions were collected every 20min into 2μL 0.01M perchloric acid. Samples consisted of three baseline fractions. Samples were stored on dry ice and then at –80°C before being analyzed. Dialysate samples were analyzed for 5-HT and the metabolite 5-hydroxyindolacetic acid (5-HIAA) content using reversed-phase high-performance liquid chromatography (HPLC) and electrochemical detection following the methods outlined previously ([Dalley et al. 2002](#_ENREF_21)) and described below. The signal was integrated using Chromeleon software (version 6.2, Dionex, United Kingdom). Because of the long-term nature of this study, for consistency, animals were dialysed in pairs (one control and one lesion, simultaneously) and their baseline dialysate samples directly compared in a single HPLC run.

**High-Performance Liquid Chromatography**

Chilled 15μL samples were separated on a C18 silica-based analytical column (100 x 4.6mm, Hypersil, Phenomenex, UK) using a mobile phase (13.6g/L KH_2_PO_4_.H2O, 185mg/L octane sulfonic acid, and 18% methanol, pH 2.75) delivered at 0.8mL/min. Levels of 5-HT, 5-HIAA, DA and NA were quantified using a dual-electrode analytical cell and electrochemical detector (Coulochem II; ESA, Chelmsford, MA) with electrode 1 set at –150mV and electrode 2 set at 180mV (5014b analytical cell; ESA) with reference to a palladium electrode. The resultant signal was integrated using Chromeleon software (version 6.2, Dionex, United Kingdom). The system was calibrated using standards containing known amounts of 5-HT, NA, and DA, with detection limits of 7.5, 2.5 and 2.5fmol, respectively. Again, because of the long-term nature of the study, HPLC analyses were performed on a batch of animals at a time, with each batch containing one control, one amygdala and one OFC lesioned animal.

**Calculation of the model parameters**

REINFORCEMENT. Each stimulus of each pair was at first assigned a notional value of 0.5 on a scale from 0–1. Following choice and reinforcement, the value of the unchosen stimulus remained unchanged but the value of the chosen stimulus was altered. In simple forms of the model, this was according to a subject-specific rate parameter *τ*, where reinforcement *R* takes the value 0 for punishment and 1 for reward:

$$\begin{matrix} \text{Prop}\left(x,a,b\right)\equiv\left(1-x\right)a+xb \\ v_{i,t+1}\leftarrow\text{Prop}\left(\tau,v_{i,t},R_{t}\right) \end{matrix}$$

This is equivalent to the one-step Q-learning process **[Watkins 1989 PhD thesis p96]** and delta-rule expressions of the same update function:

$$\begin{matrix} v_{t+1}\leftarrow\text{Prop}\left(\tau,v_{t},R_{t}\right) \\ v_{t+1}\leftarrow\left(1-\tau\right)v_{t}+\tau R_{t} \\ v_{t+1}\leftarrow v_{t}+\tau\left(R_{t}-v_{t}\right) \\ v_{t+1}\leftarrow v_{t}+\tau\delta_{R} \\ \Delta v=v_{t+1}-v_{t}=\tau\delta_{R} \end{matrix}$$

A high value of *τ* implies rapid learning from reinforcement; a low value implies slow learning and a greater dependence on prior reinforcement history (or, early on, initial values). In a more complex model, the rates of change for reward and punishment were specified separately (the *i* subscripts are omitted for clarity):

$$v_{t+1}\leftarrow\left\{\begin{matrix} \text{Prop}\left(\tau_{r},v_{t},R_{t}\right)\text{ when }R_{t}=1 \\ \text{Prop}\left(\tau_{p},v_{t},R_{t}\right)\text{ when }R_{t}=0 \end{matrix}\right\}$$

STIMULUS ‘STICKINESS’. This parameter described a subject’s tendency to persist in choosing a stimulus that it has chosen recently, regardless of reinforcement or the location of the stimulus. In models using this measure, each stimulus had a ‘stickiness’ value that began at 0. It varied according to the stimulus chosen:

$$c_{i,t+1}\leftarrow\left\{\begin{matrix} \text{Prop}\left(\tau_{c},c_{i,t},d_{c}\right)\text{ when stimulus }i\text{ chosen on trial }t \\ \text{Prop}\left(\tau_{c},c_{i,t},0\right)\text{ when not chosen/not presented} \end{matrix}\right\}$$

High values of the rate parameter *τ_c_* cause more rapid acquisition of a stimulus preference when a stimulus is chosen (regardless of reinforcement). High values of the maximum parameter *d_c_* cause a larger overall influence of stimulus ‘stickiness’, compared to the effects of reinforcement (via their relative effects upon *x*).

SIDE ‘STICKINESS’. This parameter describes a subject’s tendency to persist in choosing a side that it has chosen recently, regardless of reinforcement or the specific stimulus presented at that location. It was defined by a side-stickiness value that began at 0 and varied according to the side chosen:

$$\begin{matrix} l_{t+1}^{X}\leftarrow\left\{\begin{matrix} \text{Prop}\left(\tau_{l},l_{t}^{X},d_{l}\right)\text{ when side X chosen on trial }t \\ \text{Prop}\left(\tau_{l},l_{t}^{X},-d_{l}\right)\text{ when other side chosen} \end{matrix}\right\} \\ l_{i,t}=\left\{\begin{matrix} l_{t}^{\text{left}}\text{ when stimulus }i\text{ presented on left side on trial }t \\ l_{t}^{\text{right}}\text{ when stimulus }i\text{ presented on right side on trial }t \end{matrix}\right\} \end{matrix}$$

High values of the rate parameter *τ_l_* cause more rapid acquisition of a side preference when a side is chosen. High values of the maximum parameter *d_l_* cause a larger overall influence of side ‘stickiness’, compared to the effects of reinforcement (via their relative effects upon *x*).

**Results**

**Model Comparison**

The eight models **(Table S1)** were compared using data from all postoperative discriminations, D5–D8. The best was a model using a single reinforcement parameter (*τ*), plus stimulus-stickiness and side-stickiness parameters. (For verification, this model also won when all postoperative data, not just discriminations, were included. Additionally, it won as judged by the corrected Akaike information criterion, AIC_c_ = [ 2 *k* – 2 *LL* ] + [ 2 *k* (*k* + 1) / (*n* – *k* – 1) ].)

**Table S1**

| Arbitrary model letter | Parameters per subject, k/s | Single or double-parameter reinforcement model | Stimulus stickiness? | Side stickiness? | BIC (lower is better, * winner) |
| --- | --- | --- | --- | --- | --- |
| A | 1 | single | N | N | 13,216 |
| B | 2 | double | N | N | 13,292 |
| C | 3 | single | Y | N | 11,619 |
| D | 4 | double | Y | N | 11,737 |
| E | 3 | single | N | Y | 11,811 |
| F | 4 | double | N | Y | 11,919 |
| **G** | **5** | **single** | **Y** | **Y** | **10,120 (*)** |
| H | 6 | double | Y | Y | 10,235 |

**Necessity and sufficiency of parameter changes for behavioural effects**

To establish the necessity and sufficiency of model parameter changes to cause these behavioural effects (changes in errors to criterion and changes in obey probabilities), multiple (*n* = 1,000) virtual subjects per group were simulated (as described in main text), using the best model, this time under the following conditions:

*All varying*

(a) with all virtual subjects taking their group mean value for each parameter (thus, all parameters varied between groups);

*One parameter set varying*

(b) only the reinforcement rate parameter (*τ*) varied between groups, with all other parameters taking the overall mean values;

(c) only the stimulus stickiness parameters (*τ_c_* and *c*) varied between groups, with all other parameters taking the overall mean values;

(d) only the side stickiness parameters (*τ_l_* and *d*_l_) varied between groups, with all other parameters taking the overall mean values (the mean across both groups);

*Two parameter sets varying*

(e) the reinforcement rate and stimulus stickiness parameters (*τ, τ_c_*, *c*) varied between groups, with the side stickiness parameter taking the overall mean values.

(f) the reinforcement rate and side stickiness parameters (*τ*, *τ_l_*, *d*_l_) varied between groups, with the stimulus stickiness parameter taking the overall mean values;

(g) the stimulus-stickiness and side stickiness parameters (*τ_c_*, *c*, *τ_l_*, *d*_l_) varied between groups, with the reinforcement rate parameter taking the overall mean values.

All these conditions except (d) predicted more errors to criterion in the OFC group compared to controls, and all except (c), (d), and (g) predicted more errors to criterion in the amygdala group compared to controls.

All except (d) and (g) predicted a lower *P*(obey | ‘true’ feedback) and a higher *P*(obey | ‘false’ feedback) in the OFC group compared to controls, and all except (c), (d), and (g) predicted these effects in the amygdala group compared to controls.

Thus, the simple behavioural differences were captured by conditions (a), (b), (e), and (f), and additionally by condition (c) for the OFC group, indicating that between-group variation in the reinforcement rate was necessary and sufficient to explain these observed behavioural effects in the 5-HT-depleted amygdala group, and sufficient to explain them in the 5-HT-depleted OFC group.

Refs

Watkins CJCH 1989. Learning from delayed rewards. Unpublished PhD thesis, University of Cambridge.

**Table S1, *Post mortem* brain monoamine levels.**

| A | ***Serotonin*** | | | | | | | | |
| --- | --- | --- | --- | --- | --- | --- | --- | --- | --- |
|  | **Control** | | | **5,7-DHT OFC**  **% of control** | | | **5,7-DHT Amyg**  **% of control** | | |
| **OFC** | 0.43 | ± | 0.06 | **61.0** | **±** | **6.36*** | 123.1 | ± | 14.60 |
| mPFC | 0.57 | ± | 0.06 | 95.9 | ± | 12.38 | 108.9 | ± | 12.84 |
| vlPFC | 0.95 | ± | 0.49 | 94.5 | ± | 57.58 | 67.6 | ± | 25.12 |
| B8 | 0.36 | ± | 0.05 | 118.5 | ± | 12.57 | 91.9 | ± | 12.03 |
| B4-6 | 0.37 | ± | 0.04 | 106.6 | ± | 10.50 | 94.7 | ± | 19.58 |
| C1 | 0.46 | ± | 0.09 | 118.2 | ± | 21.20 | 108.6 | ± | 27.83 |
| **AMYG** | 1.53 | **±** | 0.24 | 94.4 | **±** | 21.01 | **64.5** | **±** | **14.5†** |

| B | ***Dopamine*** | | | | | | | | | ***Noradrenaline*** | | | | | | | | |
| --- | --- | --- | --- | --- | --- | --- | --- | --- | --- | --- | --- | --- | --- | --- | --- | --- | --- | --- |
|  | **Control** | | | **5,7DHT OFC**  **% of control** | | | **5,7-DHT AMYG**  **% of control** | | | **Control** | | | **5,7-DHT OFC**  **% of control** | | | **5,7-DHT AMYG**  **% of control** | | |
| **OFC** | 0.27 | ± | 0.02 | 117.8 | ± | 25.8 | 91.6 | ± | 14.3 | 0.49 | ± | 0.04 | 96.2 | ± | 19.6 | 139.9 | ± | 39.9 |
| mPFC | 0.30 | ± | 0.03 | 118.8 | ± | 27.1 | 98.1 | ± | 17.4 | 0.73 | ± | 0.05 | 85.7 | ± | 9.1 | 107.9 | ± | 11.4 |
| vlPFC | 0.27 | ± | 0.03 | 91.3 | ± | 14.0 | 101.0 | ± | 36.4 | 0.62 | ± | 0.08 | 121.0 | ± | 43.9 | 143 | ± | 27.2 |
| B8 | 0.52 | ± | 0.08 | 70.6 | ± | 10.9 | 68.5 | ± | 10.8 | 0.87 | ± | 0.03 | 102.1 | ± | 20.3 | 113.0 | ± | 16.1 |
| B4-6 | 0.44 | ± | 0.10 | 108 | ± | 20.5 | 94.4 | ± | 16.1 | 1.11 | ± | 0.05 | 121.1 | ± | 7.8 | 102.6 | ± | 13.5 |
| aCING | 0.37 | ± | 0.09 | 103.4 | ± | 11.3 | 83.2 | ± | 11.0 | 1.17 | ± | 0.10 | 118.3 | ± | 20.0 | 83.3 | ± | 9.2 |
| **AMYG** | 6.34 | **±** | 2.51 | 71.5 | **±** | 44.3 | 86.3 | **±** | 31.9 | 0.98 | ± | 0.21 | 75.7 | ± | 5.4 | 91.3 | ± | 12.3 |

Mean levels (pmol/mg tissue weight ± SEM) of the control group and percentage of mean control levels in marmosets with 5,7-DHT infusions into the OFC and amygdala for (A) 5-HT and (B) DA and NA. *p≤0.05, †p=0.09, compared with control group (*post-hoc* LSD test). mPFC, medial PFC; vlPFC, ventrolateral PFC; B8, Brodmann’s area 8; B4-6, motor and premotor cortex; aCING, anterior cingulate cortex; AMYG, amygdala.
